# Redefining Vaginal Agenesis Management: A Comprehensive Review

**DOI:** 10.7759/cureus.76366

**Published:** 2024-12-25

**Authors:** Prathibha Saravanakumar, Divyabharathi Selvam, Anusha K S, Uma Maheswari, Karthigeyan Jeyapalan

**Affiliations:** 1 Prosthodontics, Sri Ramachandra Institute of Higher Education and Research, Chennai, IND; 2 Prosthodontics, SRM Dental College, Chennai, IND

**Keywords:** androgen-insensitivity syndrome, custom stents, mayer-rokitansky-küster-hauser syndrome, multidisciplinary care approach, non-surgical treatments, oral and maxillofacial prostheses, prosthodontics, vaginal agenesis, vaginal dilators, vaginoplasty

## Abstract

Vaginal agenesis, a rare and complex congenital anomaly predominantly linked to Mayer-Rokitansky-Küster-Hauser (MRKH) syndrome or complete androgen insensitivity syndrome (CAIS), demands innovative and highly individualized treatment strategies to achieve anatomical and functional restoration. While non-surgical options like vaginal dilation remain foundational, the advent of custom-made stents has redefined the paradigm of care, emerging as a transformative tool in both post-surgical and non-surgical management. Bridging the expertise of prosthodontics and gynecology, personalized stents not only enhance healing and maintain patency but also elevate patient comfort and compliance. Prosthodontists, leveraging advanced material science and precision design, play a critical role in crafting bespoke stents tailored to anatomical and therapeutic needs. This review highlights the synergistic relationship between prosthodontics and gynecology, emphasizing the cutting-edge role of custom stents in improving outcomes for patients with vaginal agenesis.

## Introduction and background

Vaginal agenesis, a rare congenital anomaly characterized by the absence or underdevelopment of the vaginal canal, presents significant physical, emotional, and psychological challenges for affected individuals [1]. It is frequently associated with conditions such as Mayer-Rokitansky-Küster-Hauser (MRKH) syndrome and androgen insensitivity syndrome (AIS). With an estimated incidence of one in 4,500 female births, this condition profoundly impacts reproductive and sexual health, necessitating comprehensive, multidisciplinary intervention [2]. The primary objectives of treatment are to establish a functional vaginal canal for normal sexual function and to address the emotional and psychological well-being of patients [3,4].

Historically, treatment modalities have spanned surgical and non-surgical approaches, including vaginoplasty and the use of vaginal dilators. More recently, prosthodontists have emerged as key contributors in the management of vaginal agenesis through the design and fabrication of custom vaginal stents. These specialists, traditionally focused on oral and maxillofacial prostheses, possess unique expertise in creating patient-specific devices tailored to individual anatomical and functional needs. By leveraging advancements in biomaterials and digital technology, prosthodontists can design stents that ensure biocompatibility, comfort, and precision.

Custom vaginal stents serve a dual role in the management of vaginal agenesis. They function as dilators in non-surgical methods, gradually aiding in the formation or expansion of the vaginal canal, and as supports in post-surgical care, maintaining the patency of neovaginas and preventing stenosis during healing [5]. The utility of stents has made them an integral part of both conservative and surgical treatment strategies.

MRKH syndrome, a condition resulting from embryological failure in the development of Müllerian ducts, accounts for a significant proportion of vaginal agenesis cases. This syndrome is characterized by aplasia or hypoplasia of the vagina, uterus, and fallopian tubes [6]. Emerging research suggests that genetic dysregulation involving Wnt and Hox genes, as well as prenatal exposure to environmental stressors, may underlie these developmental anomalies. Interestingly, mechanisms associated with MRKH syndrome share similarities with those implicated in endometriosis, including aberrant Müllerian remnants, altered immune responses, and dysregulated signaling pathways [7].

Patients with MRKH syndrome often present with primary amenorrhea despite normal secondary sexual characteristics and a 46, XX karyotype. Difficulty with vaginal penetration, despite intact sexual desire and pleasure, is another hallmark symptom [8]. Conservative treatments, such as vaginal dilators, are considered first-line therapy due to their high success rates, minimal complications, and low cost. These methods offer a non-invasive alternative to surgery and empower patients to maintain greater control over their treatment [9].

In this context, custom vaginal stents represent an innovative and patient-centered advancement. By integrating expertise in prosthodontics with a multidisciplinary approach, these devices provide a promising solution for enhancing the quality of life in individuals affected by vaginal agenesis.

Thus, this study reviews the critical aspects of managing vaginal agenesis, with a focus on the design, fabrication, and application of custom-made vaginal stents. It explores the evolving role of prosthodontists in providing individualized solutions, examines the multidisciplinary collaboration required for optimal patient outcomes, and evaluates the integration of materials and digital technologies in stent development.

## Review

Classification of vaginal agenesis

From a surgical management perspective, anomalies associated with vaginal agenesis are generally categorized as follows [10]: (a) partial agenesis, characterized by a functioning midline uterus and cervix, requiring surgical intervention to create a vaginal canal for menstrual discharge, with normal pregnancy possible; (b) complete agenesis with a functioning uterine corpus but without a cervix, necessitating a hysterectomy to prevent endometriosis followed by vaginoplasty to form a functional vaginal canal; (c) complete agenesis with a rudimentary uterine bulb and a functioning endometrium, requiring abdominal surgery to remove the uterine bulb and vaginoplasty to create a neovagina; and (d) complete agenesis with a rudimentary uterine bulb but without a functioning endometrium, where vaginoplasty alone is performed to establish a functional vaginal canal.

This classification aids in determining the appropriate surgical approach based on the specific anatomical and functional features of the uterine and vaginal structures.

Evolution of vaginal dilators

**Figure 1:**
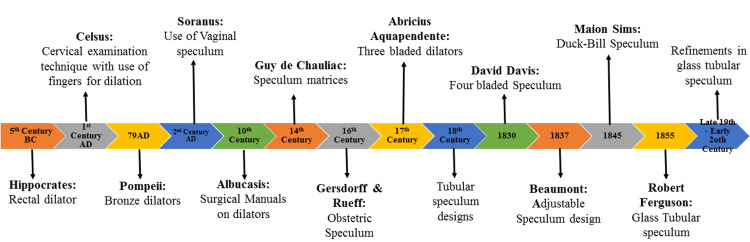
Evolution of vaginal dilators Image Credits: Dr. Prathibha Saravanakumar

Figure 1 represents a historical timeline highlighting the evolution of cervical and vaginal dilators from the 5th century BC to the early 20th century [11].

Various treatment modalities for vaginal agenesis

Management of vaginal agenesis varies globally, reflecting differences in cultural, medical, and healthcare priorities.

Vaginal Dilation

Vaginal dilation therapy, first described by Frank in 1938 [12] remains the preferred first-line treatment in the UK and the US. This method employs vaginal molds of increasing width and length to stretch the vaginal dimple, creating a functional vaginal canal. Patients apply gentle pressure for at least 30 minutes daily over several months, aiming to achieve a vaginal length of 7-8 cm. Success rates can reach up to 90%, particularly when the therapy is delivered by a multidisciplinary team providing psychological support.

While vaginal dilation is non-invasive and cost-effective, compliance and satisfaction rates are low due to the lengthy, often painful process, and the emotional reminder of abnormality. Psychological support is critical for maintaining motivation. Additionally, dilation therapy may still be required after surgical reconstruction to maintain vaginal patency. Timing should consider the patient's emotional maturity, and the option of choosing between dilation and surgery should be individualized [13,14].

Vaginal Dilators

Vaginal dilators are smooth, cylindrical devices available in progressively larger diameters. They facilitate tissue stretching, pelvic floor muscle relaxation, and desensitization. However, poor compliance, pain, and discomfort remain significant barriers. Adherence can improve with clear therapeutic goals, supportive supervision, and a multidisciplinary approach to managing fear and anxiety [15-19].

Surgical Vaginal Reconstruction

Surgical techniques for vaginal agenesis vary widely, with some European countries preferring surgery as the first-line approach, often followed by postoperative dilation therapy. Common surgical methods include: 

Pressure method (Vecchietti procedure): The Vecchietti technique creates a neovagina by gradually stretching the patient’s vaginal skin using a traction device. The procedure involves placing an olive-shaped bead on the vaginal dimple, secured by threads passed subperitoneally through the vesicorectal space and out through the abdomen, where they attach to a suprapubic traction device. Initially, the bladder is catheterized, and pneumoperitoneum is achieved via a transumbilical approach. A 10-mm laparoscope and two 5-mm lateral trocars are inserted to guide the procedure, and a rectal probe outlines the rectum. The vesicorectal space is dissected, and the bladder is reflected anteriorly. Using a Vecchietti straight needle, the threads are passed subperitoneally into the vesicorectal space under direct visualization. The olive bead is secured externally at the vaginal dimple, while the threads are pulled through the abdominal wall and connected to the traction device. The peritoneum is closed with absorbable suture [20-22].

Neovagina creation (Davydov procedure): The modified laparoscopic Davydov technique creates a neovagina in two steps: laparoscopic and vaginal. During the laparoscopic step, the pelvic peritoneum is mobilized to form the neovaginal walls. The peritoneum beneath the uterine remnants is incised, and the round ligaments are cut bilaterally. Purse-string sutures are placed to secure the peritoneal edges to surrounding structures, ensuring mobilization for the neovagina. In the vaginal step, an H-shaped incision is made on the vaginal vestibulum, and blunt and sharp dissection separates the bladder and rectum, exposing the mobilized peritoneal margins. These margins are sutured to the vaginal mucosa, and a paraffin gauze dressing is placed in the neovagina [23].

McIndoe reed vaginoplasty: The McIndoe procedure creates a neovagina by forming a vesicorectal space through a small incision at the fourchette, followed by blunt dissection to achieve a length of 10-12 cm. The space is lined with a split-thickness skin graft, typically harvested from the buttock, thigh, or suprapubic region, or occasionally an amnion graft. The graft is sewn onto a mold, with its deep surface outward, and inserted into the cavity. A mold, either rigid or malleable, is used to maintain the neovagina’s shape, with the graft edges sutured to the perineal incision, forming the introitus. Traditionally, the mold remains in place for up to three months to prevent contraction. This technique, pioneered by Abbe and refined by McIndoe and others, remains a foundational approach for vaginal reconstruction [24].

Williams vulvovaginoplasty: The William vulvovaginoplasty involves creating a neovagina through a horseshoe-shaped vulvar incision made lateral to the midline and extending above the urethral orifice, with an indwelling bladder catheter in place. The incision base curves gently across the fourchette. The edges are freed by gentle undercutting, and the internal edges are sutured together in the midline with knots positioned inside the vaginal lumen. The internal skin margins are then approximated with interrupted sutures. The resulting pouch allows the insertion of two fingers to a depth of approximately 3 cm. Colon vaginoplasty procedure utilizes a segment of the rectosigmoid colon to line the neovaginal space instead of a split-thickness skin graft, following the creation of a vaginal cavity in a manner similar to the McIndoe operation [25].

Laparoscopic advancements have improved the safety and efficacy of all these procedures, excluding the Williams and McIndoe Reed approaches [26,27].

Timing of treatment

Intervening in vaginal agenesis is ideally deferred until adolescence or early adulthood, aligning with the patient’s attainment of physical and psychological maturity. Early procedures, historically performed during infancy or prepubescence, have fallen out of favor due to their lack of functional necessity and frequent requirement for surgical revisions before sexual activity begins. Delaying intervention allows the individual to be directly involved in their care decisions, fostering autonomy and enhancing adherence to essential post-treatment protocols [28]. 

Patient engagement is paramount, particularly in the success of vaginal dilation therapy, whether employed as a standalone approach or adjunctive to surgery. Initiating dilation during adolescence or later significantly improves compliance and outcomes, as it necessitates consistent effort to prevent postoperative stenosis and ensure functional restoration [13,29].

In complete androgen insensitivity syndrome (CAIS), the timing of gonadectomy introduces additional complexities. Although the risk of malignancy is negligible before puberty, deferring gonadal removal until post-puberty enables natural pubertal development driven by testosterone-to-estradiol conversion. This delay also provides the patient with the necessary time to assimilate their diagnosis and participate meaningfully in treatment planning [13].

Expertise in anatomical replication

Prosthodontists, traditionally specializing in oral and maxillofacial prosthetics, play a vital role in managing vaginal agenesis through their expertise in designing and fabricating custom vaginal stents. Their skills in materials science, biocompatibility, and precision device fabrication are integral to creating stents that conform to the unique dimensions and contours of the vaginal canal. Leveraging advanced impression techniques or 3D imaging, prosthodontists ensure optimal fit and comfort using materials such as medical-grade silicone or thermoplastic elastomers, known for their flexibility, durability, and biocompatibility. 

Collaboration with gynecologists is central to their approach, with joint consultations enabling precise customization based on surgical outcomes and patient feedback. Prosthodontists refine stent dimensions and surface properties to align with gynecological requirements, ensuring the stents promote healing and maintenance. Their patient-centric approach prioritizes adjustments in stent design to enhance ease of use, accommodate individual preferences, and support psychosocial comfort. Follow-up care ensures modifications address anatomical changes during healing, further optimizing outcomes.

Custom-made stents offer distinct advantages over prefabricated alternatives, including anatomical precision that enhances comfort and compliance, designs that promote even pressure distribution to support epithelialization and healing, and personalized features that minimize vaginal stenosis. These factors contribute to sustained functional outcomes and improved sexual satisfaction, underscoring the critical contributions of prosthodontists in this multidisciplinary treatment approach.

Materials used

Dental materials play a vital role in vaginal stent fabrication due to their unique properties such as precision, biocompatibility, and ease of manipulation. These attributes facilitate the creation of customized, patient-specific stents that ensure anatomical accuracy, comfort, and long-term functionality [30].

Impression Materials

Dental impression materials are essential for capturing accurate molds of the vaginal canal. The dental impression compound is widely used for initial impressions. Its thermoplastic nature allows it to be shaped easily when heated and hardened when cooled, making it a safe and practical choice for reusable molds. For more intricate anatomical details, polyvinyl siloxane (PVS) offers superior dimensional stability and tear resistance. This advanced elastomeric material ensures precise impressions, critical for stents requiring a custom and comfortable fit. 

Casting Materials

Casting materials provide the foundation for creating molds or models during stent fabrication. Dental plaster is a quick-setting and easy-to-use material, suitable for temporary molds in preliminary design phases. However, it lacks the durability required for long-term applications. In contrast, dental stone provides greater strength and precision, making it ideal for creating stable and accurate molds for custom stents. Die stone, known for its high durability and dimensional stability, is preferred for detailed and long-lasting molds that demand exact replication of anatomical structures. 

Patterning Material

Modeling wax is highly valued in the design and customization of vaginal stents. Its pliability allows for precise modifications, ensuring that stents can be tailored to fit specific anatomical shapes. This material is particularly useful during the prototype stage, facilitating adjustments before final production. 

Stent Materials

The choice of stent material significantly impacts the durability and comfort of the final product. Cold-cure acrylic resin is ideal for temporary or short-term stents due to its ease of manipulation and self-curing properties. For long-term solutions, heat-cure acrylic resin offers superior strength, biocompatibility, and resistance to deformation, ensuring prolonged use with minimal risk of irritation. Silicone elastomer, with its softness, flexibility, and tissue compatibility, is highly suitable for stents designed for extended wear, providing exceptional patient comfort and maintaining functionality over time. 

Fabrication techniques for vaginal stents

Several methods have been described for the fabrication of custom vaginal stents, emphasizing precision, comfort, and clinical efficacy. 

MRI-Based Dimensional Planning

Dimensions are determined using MRI reports, with stents designed to be 3 mm smaller than the planned diameter [31]. A cylindrical mold is created using a wax-modified syringe tip, followed by an alginate impression poured with type III dental stone. Subsequent steps involve creating a PVS mold reinforced with stainless steel wire to ensure durability. The mold is invested, dewaxed, and packed with heat-activated acrylic resin. A hollow design is achieved by removing the putty material, reducing weight for enhanced patient comfort. 

3D Printing Technology

Using CAD software, applicator designs are based on physical examinations and imaging [32]. Surface channels for interstitial needles or central catheters are incorporated as needed. Thermoplastic materials like PC-ISO, known for their biocompatibility and sterilizability, are employed. Prototypes undergo rigorous quality assurance to ensure clinical suitability, with reported benefits including optimal dose distribution in brachytherapy applications. 

Custom Acrylic Tray

A cylindrical custom tray, 3-4 mm smaller than the vaginal cavity dimensions, is fabricated using cold-cure acrylic resin [33]. Impressions are made with rigid materials such as impression compounds, ensuring precise replication of the neovaginal dimensions. The stent is then fabricated in two halves using a dental plaster mold, incorporating a hollow design for lightness and drainage. The final stent is polished and delivered with patient-specific hygiene and usage instructions. 

Each technique emphasizes patient-centric outcomes, such as anatomical precision, lightweight, and comfort, ensuring clinical effectiveness and sustained therapeutic results.

Various studies

Table 1 provides a comprehensive overview of various materials and fabrication techniques utilized in the design and development of vaginal stents for the management of vaginal agenesis.

**Table 1:** Overview of vaginal stent fabrication techniques, materials, and clinical outcomes in the management of vaginal agenesis: insights from various studies PVS, polyvinyl siloxane; PLA, polylactic acid; PVC, polyvinyl chloride; TVL, total vaginal length

S. No.	Author	Study design	Material used	Fabrication technique	Clinical outcome
1	Coskun A et al. (2007) [34]	Case report	Skin graft, silicone-coated stent	Full/split-thickness graft with silicone-coated prosthesis used for McIndoe technique.	Maintained vaginal depth with minimal granulation; satisfactory healing and sexual activity without complications.
2	Rathee M et al. (2014) [35]	Case report	Chemically cured acrylic resin	Wax scaffold layered with acrylic resin, dewaxed for hollow structure, and polished.	Simple, affordable stent (9×2×2.5 cm) prevented contracture post-vaginoplasty. Persistent use resulted in a functional neovagina.
3	Kamalakannan J et al. (2015) [36]	Case report	Silicone elastomer	RTV silicone stent fabricated from impressions, designed for comfort and ease of insertion.	Lightweight, comfortable stent improved patient compliance and overall quality of life post-vaginoplasty.
4	Mishra B et al. (2016) [37]	Prospective case series	Dental impression compound, acrylic	Modified vaginal mold fabricated from a 10 mL disposable syringe, used for graft application and dilation.	All patients achieved a vaginal depth of 9-10 cm within six months. There were no major complications, such as graft loss, mold extrusion, stenosis, or fistulas. Additionally, uterine drainage was successfully achieved in all four patients who required it.
5	Ogliari KC et al. (2020) [9]	Case report	Modeling wax, heat-cure acrylic resin, silicone	Wax pattern processed with acrylic; salt used for hollowing and self-cure acrylic for sealing. Silicone dilators also used for dilation.	Neovagina formed with minimum dimensions achieved. Both cases had successful outcomes with no complications and comfortable sexual activity reported.
6	Mohamed K et al. (2021) [38]	Case report	Heat-cure acrylic resin, self-cure resin	Cylindrical prosthesis hollowed using wax and processed with acrylic resin; modified with orthocal gypsum and self-cure resin.	Regular use increased vaginal dilation, with progressive improvement in prosthesis size and patient adherence.
7	Belkhode VM et al. (2021) [31]	Case report	Acrylic resin	Hollow stent fabricated with heat-cure acrylic using ice as a core material; hole sealed with self-cure acrylic.	Successfully maintained neovaginal cavity, preventing fibrosis. Patient adhered to usage instructions with no complications during healing.
8	Fernandes MS et al. (2022) [39]	Observational (non-randomized, non-clinical trial)	PLA	Custom 3D-printed PLA dilators (1.5x8 cm, 2x9 cm, 2.5x12 cm) were designed using AutoCAD and FreeCAD. Patients used the dilators with condoms for hygiene and performed gradual dilation exercises, with monthly evaluations over two years. A target TVL of 6 cm was set to enable sexual intercourse.	The average initial TVL was 1.81 cm, which increased to 6.37 cm following treatment. 14 out of 16 patients (87.5%) achieved a TVL of ≥6 cm within an average of 5.6 months. No adverse effects were reported, aside from two cases of inadvertent urethral dilation, which were resolved through reorientation of the dilators.
9	Raza FB et al. (2023) [40]	Case report	PVC tube, wax, heat-cure acrylic, elastomeric material	PVC-based stent processed with heat-cure acrylic and elastic chains for retention, wax added for dimension adjustment.	Customizable, cost-effective stent provided successful vaginal dilation with patient compliance and satisfaction over a two-year follow-up.
10	Boomiraj R et al. (2023) [41]	Case report	Heat-cure acrylic, salt	Wax pattern invested, cured with heat-cure acrylic, and hollowed with salt for prosthesis.	Stable prosthesis supported tissue healing and hygiene. Patient was satisfied with minimal need for modifications.
11	Khubchandani SR et al. (2023) [42]	Case report	Cold-cure acrylic, PVS	Two-piece mold fabricated with ice for hollowing; perforated acrylic stent finished and polished.	Effective maintenance of neovaginal patency and tissue healing with stent insertion. Patient followed instructions and achieved desired outcomes.
12	Beri A et al. (2023) [43]	Case report	3D printing resin, acrylic resin, amniotic membrane	3D-printed resin stent and conventional acrylic stent; amniotic membrane wrapped for postoperative maintenance.	Gradual adaptation ensured functional neovagina formation. Follow-up ensured patient compliance and positive outcomes with effective vaginal dilation.
13	Lim CK et al. (2024) [44]	Case report	Alginate impression material, silicone	Measured vaginal length and diameter using a condom filled with alginate. Initially fabricated a customized 3D-printed vaginal mold using silicone and periodically recreated to match evolving vaginal dimensions.	Postoperative assessment showed no vaginal stenosis. The patient resumed normal activities without discomfort.
14	Chen PH et al. (2024) [45]	Case report	Silicone polymer	Liquid silicone was poured into the mold to fabricate the sleeve. Assembly involved aligning the inner rod within the silicone sleeve. The chamber is pressurized with air, enabling the sleeve to expand during operation.	Soft, flexible, and reversible expansion minimizes discomfort and is suitable for treating vaginal stenosis.
15	Bhalerao KV et al. (2024) [46]	Case report	Impression compound, thigh skin graft	Prosthesis made from impression compound and skin graft; tailored to postoperative requirements.	Patient complied well with stent usage, achieving successful neovaginal cavity creation and minimal contraction.
16	Wilson T et al. (2024) [47]	Case report	Wax, alginate (Zhermack Hydrogum 5), type III dental stone, PVS putty, stainless steel wire, heat-activated acrylic resin (DPI Heat Cure), auto polymerizing acrylic resin (DPI RR cold cure)	An alginate impression is taken and poured with dental stone to create a cast. PVS putty is molded around twisted stainless steel wire to form a hollow mold. Heat-activated acrylic resin is used for stent fabrication. The wax pattern is invested, dewaxed, and packed with resin via compression molding. The stainless steel wire ensures a hollow, lightweight design. The stent's open end is sealed with cold-cure resin, and the surface is polished for safety.	Hollow, lightweight acrylic vaginal stent customized to patient dimensions ensures comfort and reduces fungal infection risk. Durable and economical stents prevent stricture and contraction post-surgery, offering superior patient compliance. Effective in maintaining neovaginal patency postoperatively with minimal risk of fungal infections or discomfort.
17	Gorripati JP et al. (2024) [48]	Case report	Clear heat-cure acrylic resin, frozen coconut oil	Hollow stent made by incorporating frozen coconut oil during acrylic packing, dewaxed, finished, and polished. Mold slightly smaller than neovagina dimensions.	Cost-effective, easy-to-fabricate stent effectively maintained postoperative vaginal patency, aiding successful management of vaginal agenesis.
18	Ansar H et al. (2024) [49]	Case report	Self-polymerizing acrylic resin	Mold created with wax sheets and resin packed for prosthesis; polished to prevent irritation.	Properly used dilators maintained vaginal patency and alleviated symptoms through regular use and hygiene education.

Challenges and future directions

Addressing the challenges and advancing the future of custom vaginal stents necessitate a comprehensive and interdisciplinary approach. Multidisciplinary coordination among prosthodontists, gynecologists, and psychologists remains a significant hurdle, as differing clinical priorities and workflows often complicate collaboration. Additionally, patient-specific anatomical variability and psychological considerations demand highly tailored solutions, increasing the complexity of design and fabrication. Cost and accessibility further pose barriers, as custom stents are generally more expensive than prefabricated alternatives, limiting their availability in resource-constrained settings. 

Future directions in this field hold promise for overcoming these challenges. Innovations in materials, such as bioengineered and biodegradable options, have the potential to enhance the safety, biocompatibility, and functionality of vaginal stents. The expanded use of digital technologies, including advanced scanning, CAD/CAM systems, and 3D printing, offers greater precision, efficiency, and customization in stent fabrication. Moreover, specialized training programs for prosthodontists will be essential to equip them with the skills required to address the unique demands of vaginal stent design and contribute effectively to multidisciplinary management. 

While custom-made vaginal stents offer a transformative approach to managing vaginal agenesis, their success depends on innovative, patient-centered solutions and a robust interdisciplinary framework. The inclusion of psychological support is vital to address the multifaceted needs of patients, while future advancements in technology and training will further improve accessibility and outcomes. Comparative studies focusing on long-term results, encompassing anatomical success, psychological health, emotional well-being, and sexual satisfaction, are crucial to guide evidence-based decision-making. By integrating these advancements and insights, custom vaginal stents can continue to evolve as a pivotal solution for enhancing the quality of life in individuals with vaginal agenesis.

## Conclusions

A comprehensive, multidisciplinary approach is paramount in the management of vaginal agenesis, necessitating the collaborative expertise of gynecologists, prosthodontists, and other specialists. Prosthodontists assume a critical role in the precise design and fabrication of custom vaginal stents, utilizing cutting-edge materials and digital technologies to ensure optimal anatomical compatibility and patient comfort. This integration of advanced prosthetic techniques significantly enhances both functional restoration and emotional well-being. The synergy between gynecological and prosthodontic care facilitates a highly individualized treatment plan, promoting superior outcomes, improving patient compliance, and ultimately elevating the quality of life for those affected by vaginal agenesis.
